# Modeling of Usual Care: Vasopressor Initiation for Sepsis With Hypotension

**DOI:** 10.3389/fmed.2022.715856

**Published:** 2022-03-11

**Authors:** Varesh Prasad, Andrew T. Reisner, James C. Lynch, Michael R. Filbin, Thomas Heldt

**Affiliations:** ^1^Institute for Medical Engineering and Science, Massachusetts Institute of Technology, Cambridge, MA, United States; ^2^Harvard-MIT Division of Health Sciences and Technology, Massachusetts Institute of Technology, Cambridge, MA, United States; ^3^Department of Emergency Medicine, Massachusetts General Hospital, Boston, MA, United States; ^4^Department of Electrical Engineering & Computer Science, Massachusetts Institute of Technology, Cambridge, MA, United States

**Keywords:** sepsis, septic shock, emergency medicine, mathematical modeling, usual care, vasopressors

## Abstract

Usual care regarding vasopressor initiation is ill-defined. We aimed to develop a quantitative “dynamic practice” model for usual care in the emergency department (ED) regarding the timing of vasopressor initiation in sepsis. In a retrospective study of 589 septic patients with hypotension in an urban tertiary care center ED, we developed a multi-variable model that distinguishes between patients who did and did not subsequently receive sustained (>24 h) vasopressor therapy. Candidate predictors were vital signs, intravenous fluid (IVF) volumes, laboratory measurements, and elapsed time from triage computed at timepoints leading up to the final decision timepoint of either vasopressor initiation or ED hypotension resolution without vasopressors. A model with six independently significant covariates (respiratory rate, Glasgow Coma Scale score, SBP, SpO_2_, administered IVF, and elapsed time) achieved a C-statistic of 0.78 in a held-out test set at the final decision timepoint, demonstrating the ability to reliably model usual care for vasopressor initiation for hypotensive septic patients. The included variables measured depth of hypotension, extent of disease severity and organ dysfunction. At an operating point of 90% specificity, the model identified a minority of patients (39%) more than an hour before actual vasopressor initiation, during which time a median of 2,250 (IQR 1,200–3,300) mL of IVF was administered. This single-center analysis shows the feasibility of a quantitative, objective tool for describing usual care. Dynamic practice models may help assess when management was atypical; such tools may also be useful for designing and interpreting clinical trials.

## 1. Introduction

Sepsis and septic shock represent major public health challenges, contributing to 1 in every 2 to 3 hospital deaths in the United states (1, 2). Biologically, sepsis is a dysregulated host response to infection, and septic shock is a subset of sepsis in which particularly profound circulatory, cellular, and metabolic abnormalities occur, with a greater risk of mortality than with sepsis alone (3). Clinically, septic shock is simply defined as a condition of sepsis when vasopressors are required for blood pressure support.^1^ Yet practice guidelines allow for substantial clinical practice variability regarding when vasopressors should be started to treat hypotension by initiating vasopressors—as opposed to treatment with ongoing intravenous fluid (IVF) boluses or even tolerating persistent hypotension.^2^ The definition, therefore, remains a near tautology: being treated for septic shock with vasopressors essentially fulfills the diagnostic criteria for septic shock.

In this report, we seek to define a statistical “usual care for vasopressor” (UCV) model that models clinicians' decisions about when to initiate vasopressors for hypotensive patients with sepsis. Our overarching hypothesis was that a single statistical model can match the typical management of most patients (i.e., model “usual care”) based on clinical parameters. It is worth noting that there is no current consensus about how to define this usual care. Consider the controversy related to the ongoing CLOVERS multi-center trial, which is a prospective trial comparing early vasopressors vs. a purported “usual care.” The CLOVERS investigators have assumed that the usual care for patients with sepsis and hypotension is “liberal fluids” before starting vasopressors (6). Yet critics have suggested that “liberal fluids” does not actually represent usual care; that the CLOVERS trial is, therefore, comparing two non-standard treatment strategies; and that it will be difficult to interpret the CLOVERS trial findings because the investigational strategies are not being compared directly against usual care (7). Such controversy might be addressed with an objective tool for *characterizing and quantifying* usual care in a patient population; and such a tool, the usual care provided to one population (*e.g*., the CLOVERS control group) could be directly compared against the care provided in another patient population (*e.g*., another hospital not participating in the trial, or historical controls).

There are other potential benefits to such a model. Identification of the factors associated with the transition from sepsis to septic shock may help better understand the overall pathology. As well, a dynamic practice model could provide real-time decision support, advising clinicians when current management is discordant with usual care, which might be a tool for reduced management errors.

To these ends, we developed usual care model for vasopressor initiation in hypotensive, septic Emergency Department (ED) patients. We implemented a primary model and compared its performance against alternative models to confirm the validity of the primary model. Lastly, we demonstrated how such modeling can be used for comparing different populations, and for studying outliers within a population.

## 2. Materials and Methods

**Figure 1:**
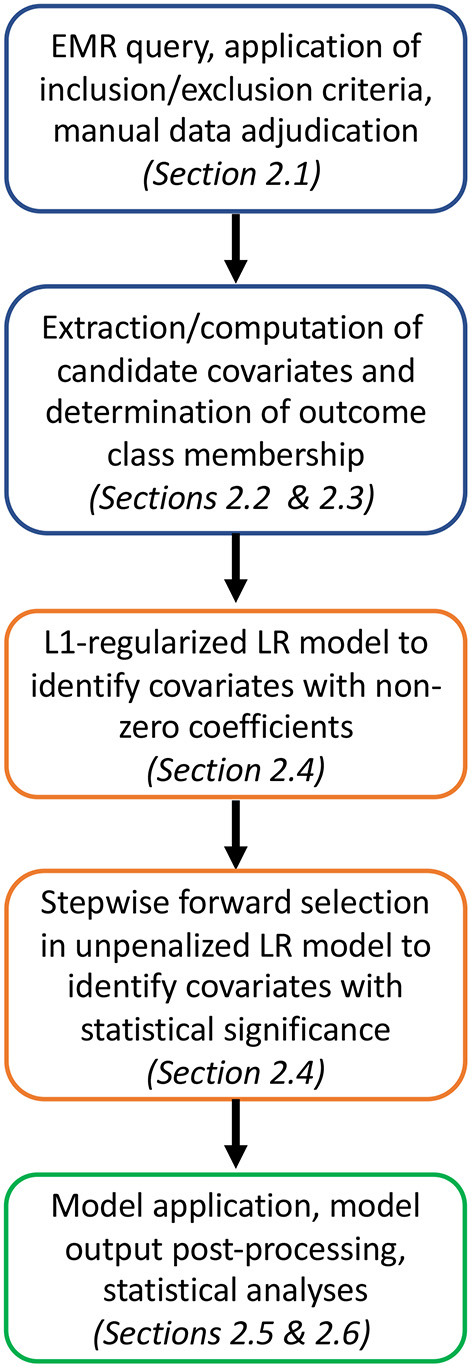
Analysis pipeline from cohort selection and candidate covariate and outcome class assignment (blue) to model training and covariate down-selection (orange) to model application, validation and statistical analyses (green).

The overall approach to developing, testing, and validating the UCV model is outlined in Figure 1.

### 2.1. Design, Setting, and Participants

**Figure 2:**
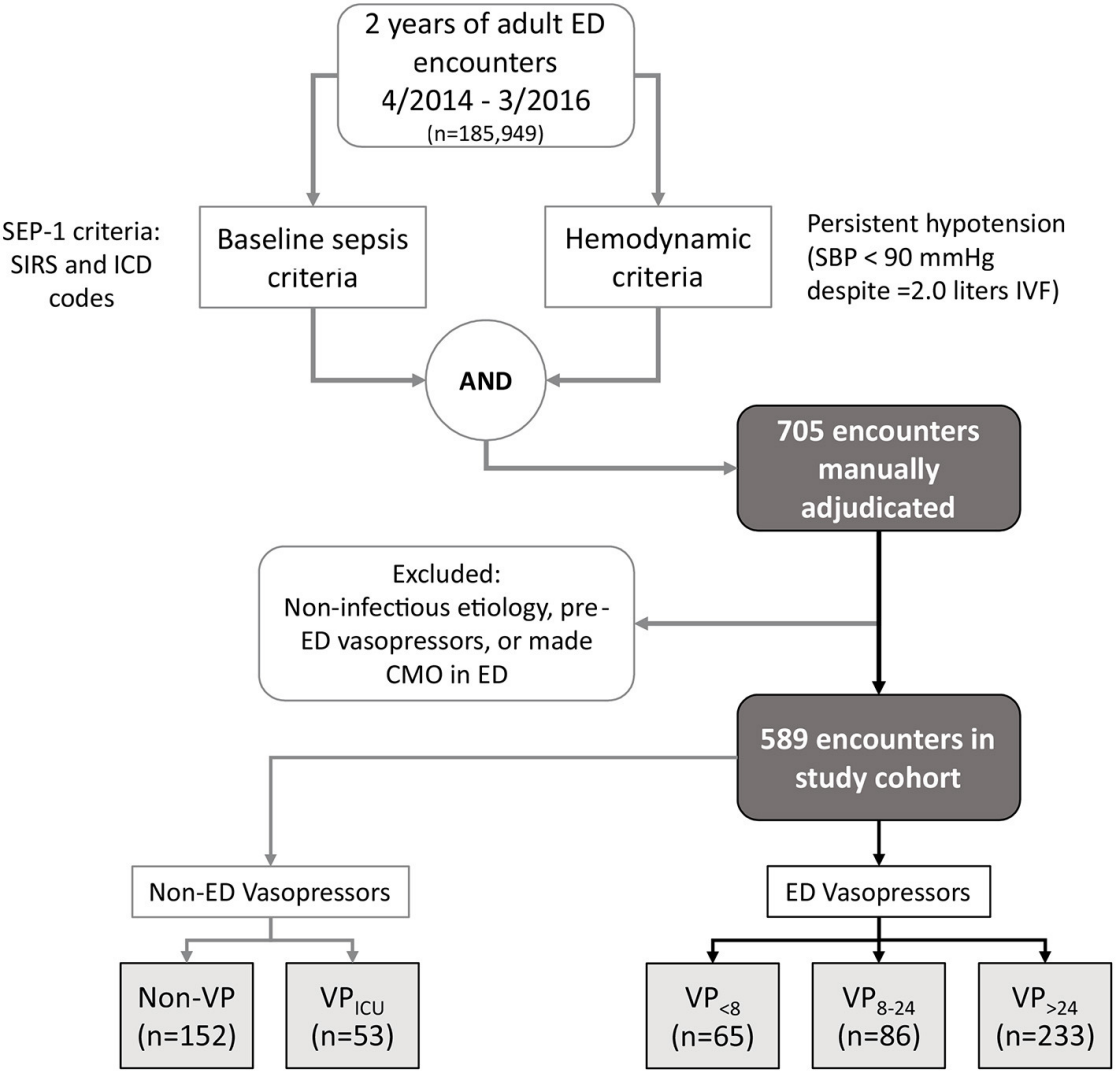
All adult ED patient encounters treated between April 2014 and March 2016 were considered for inclusion in our study. Chart review was performed on those meeting baseline sepsis and hemodynamic criteria, with subsequent exclusion of any patient i) without a likely or possible infectious etiology for ED organ dysfunction, ii) any patient managed with vasopressors in the 12 h before presenting, and iii) any patient made CMO in the ED. The study cohort was split into five mutually exclusive outcome subgroups (see text for definitions). ED, emergency department; CMO, comfort measures only; ICD, international classification of disease; IVF, intravenous fluid; SIRS, systemic inflammatory response syndrome; SBP, systolic blood pressure.

This was a retrospective analysis conducted in accordance with STROBE guidelines for cohort studies (8). With approval from the Partners Human Research Committee (Institutional Review Board Protocol # 2014P001192), we performed a *de novo* analysis of data from (9) population of all adult (age ≥ 18 years) Emergency Department (ED) patients from April 1, 2014 to March 31, 2016 who met criteria adapted from the current Centers for Medicare and Medicaid Services Severe Sepsis/Septic Shock Early Management Bundle (SEP-1) definition (10) for septic shock: (1) a final discharge diagnosis for sepsis per hospital billing codes (International Classification of Diseases [ICD], 9th or 10th edition); (2) either confirmed source of infection or high suspicion for infection documented in the admission note, and (3) development of persistent hypotension (systolic blood pressure [SBP] < 90 mmHg on at least two measurements), lactate ≥ 4.0 mmol/L, or use of vasopressors in the ED. These inclusion criteria are summarized in Figure 2. This was a secondary analysis of the population of (9), further excluding any patients that received vasopressors within 12 h prior to ED presentation or that were made comfort-measures-only in the ED. In this analysis, we only studied time points when a patient's systolic blood pressure [SBP] was below 90 mmHg, for which management options are initiation of vasopressors; administration of intravenous fluids; or further observation without intervention.^3^

### 2.2. Outcomes

We analyzed five mutually exclusive outcomes, denoted as follows:

- Non-VP: did not receive any vasopressors within 48 h of ED presentation (i.e., not in the ED nor subsequently in the hospital);
- VP_>24_: started on vasopressors in the ED, and then continued (*i.e*., in the ICU) for total course duration > 24 h; these patients were managed over multiple shifts in the ED and ICU and, therefore, multiple physicians concurred independently with the appropriateness of vasopressor use;
- VP_8−24_ and VP_ < 8_: started on vasopressors in the ED for total course durations of only 8–24 h, or < 8 h, respectively, spanning ED and any subsequent ICU care. These patients were weaned relatively quickly from vasopressors and (in theory) some may have been started on vasopressors unnecessarily; and
- VP_ICU_: did not receive ED vasopressors despite ED hypotension, but did receive vasopressors within 48 h of ED presentation (in all such cases, vasopressor initiation occurred in an ICU, or rarely, in an operating room).

### 2.3. Candidate Predictors

We analyzed the following candidate predictors: routine vital signs; age; gender; race; time elapsed in the ED; major pre-existing comorbidities (active cancer, chronic liver disease, diabetes, end-stage renal disease, immune compromise, physical disability, and congestive heart failure or any stage of chronic kidney disease); laboratory results (initial lactate, worst ED lactate, white blood cell count, creatinine); presence or absence of common symptoms of infection in groupings by organ system where possible (gastrointestinal symptoms, general fatigue or malaise, mental status change, neurological symptoms, pain, respiratory symptoms, skin findings, urinary symptoms); presentation by referral for an infectious reason.

For vital signs and lab values, we analyzed both the most recent documented value at any given time *t*, plus a weighted average using weights that decreased by half at each preceding observation (*e.g*., the third most recent observation from time *t* would have a weight that is one-eighth the weight of the observation *t*). This exponential weighting incorporates information from prior measurements while giving greater weight to more recent measurements (11). Laboratory results, vital signs, patient locations, demographics, and hospital outcome were extracted electronically from the hospital electronic data warehouse.

Some parameters required chart review: presenting symptoms, comorbidities, referral information, IVF administrations, time of first vasopressor administration, and duration of vasopressor administration. For these parameters, after training with practice charts, two trained chart reviewers independently reviewed clinical documentation (triage note, nursing and providers' notes, flowcharts) and completed a standardized data entry form (12). For every subject, the annotations of the two reviewers were compared and disagreements resolved by majority vote with a third (physician) reviewer. Cohen's Kappa was computed for reviewer-coded parameters.

### 2.4. UCV Model Development

For training the UCV model, we randomly selected 90% of VP_>24_ patients (for whom vasopressor appropriateness was corroborated by multiple sequential physicians in the ED and ICU) and 90% of Non-VP patients (for whom vasopressors were not initiated by any treating physician over two days). The VP_8−24_, VP_ <8_, and VP_ICU_ groups were considered in other analyses described below.

The UCV model was trained to data values from the “final decision time,” *t*_*f*_. Specifically, *t*_*f*_ was the last time with either: (i) documented SBP < 90 mmHg and before documented ED vasopressor initiation for VP_>24_; or (ii) SBP < 90 mmHg for Non-VP. The intent was to optimize the UCV model's ability to discriminate between conditions when clinicians definitively opted to initiate vasopressors vs. conditions when hypotension was about to conclusively resolve without vasopressors. All covariates were *z*-score standardized at *t*_*f*_. Missing values for any parameter at *t*_*f*_ were carried forward from previous times; when missing completely from the interval between ED arrival through *t*_*f*_, we used the population median of the parameter at *t*_*f*_.

A two-stage process selected variables for inclusion in a logistic regression (LR) model. We first included all covariates, as computed at *t*_*f*_, in an L1-regularized LR model. This type of regularization generates a model in which few covariates retain a non-zero coefficient (11). We used 5-fold cross-validation to select the regularization strength, maximizing the area under the receiver-operator characteristic curve (ROC AUC). Next, covariates with non-zero regression coefficients were entered into a stepwise forward selection process to select only those retaining significance (*P* < 0.05) in a final multi-variate LR model. The output from the *t*_*f*_ LR model (hereafter termed the “*the UCV model score”*) is an estimated empirical probability that an observation is from a VP_>24_ patient. With this final model, we computed the ROC AUC for *t*_*f*_ under leave-one-out cross-validation for the training set, and also applied it to the entire validation cohort (10% of VP_>24_ patients and Non-VP patients).

*Comparison with alternative models*—To determine whether the UCV model performance holds up at time points prior to *t*_*f*_, we applied the UCV model to earlier time points, and compared its performance with that of alternative models that were trained specifically to those earlier time points. The goal was to determine whether alternative clinical parameters or different parameter weights might provide significantly better discrimination at time points before *t*_*f*_:

- To develop the alternative models, we used the same methodology as above, but trained models on data at *t*_*f*−1_, *t*_*f*−2_, *t*_*f*−3_, and *t*_*f*−4_, with *t*_*f*−1_ being the time of observation of the set of vital signs documented immediately prior to the observation at *t*_*f*_, *t*_*f*−2_ being the time of observation of the vital signs immediately prior to those at *t*_*f*−1_, and so on. We excluded data from time points with SBP ≥ 90 mmHg, and used the same class labels.
- We applied the UCV model to preceding sets of documented vital signs. Likewise, we applied the *t*_*f*−1_, *t*_*f*−2_, *t*_*f*−3_, and *t*_*f*−4_ models for comparison.

We compared the ROC AUCs for the training cohort (computed by leave-one-out cross-validation) to test whether the alternative models were significantly better than *t*_*f*_ for earlier observations.

### 2.5. UCV Model Applications

*Assessment of UCV model:* We computed the ROC AUCs for the training cohort (using leave-one-out cross validation) and for the testing cohorts, applying the model to all hypotensive time points. This provided a general assessment of the UCV model to predict clinicians' decisions, i.e., how well usual care was modeled throughout the ED stays.

*Application of the UCV model to compare care between different populations #1:* We explored how dynamic modeling can be used for hypothesis testing, i.e., comparing the care in different populations/cohorts. We hypothesized that septic patients who were quickly (≤ 24 h) weaned from ED vasopressors had care that significantly different from usual care; instead, this population had vasopressors initiated more liberally (and potentially, unnecessarily). To test these hypotheses, we applied the UCV model to compare care between different study cohorts. To test the first hypothesis, we computed the UCV model scores for the cohorts VP_8−24_ and VP_ <8_ at *t*_*f*_. We compared the distributions of scores for these groups to the distribution for the VP_>24_ group. If the VP_8−24_ or VP_ <8_ groups had significantly lower model scores compared to the VP_>24_ group, we would take this as evidence of vasopressors having been started outside of the usual care (and potentially unnecessarily).

*Application of the UCV model to compare care between different populations #2:* We further hypothesized that septic patients who did not have vasopressors started in the ED but did have vasopressors started shortly after ICU admission had care that was significantly different from usual care; instead, this population had delayed vasopressor initiation relative to the usual care. To test this second hypothesis, we compared distributions for the VP_ICU_ group with the Non-VP group at *t*_*f*_ (the time of the last ED SBP < 90 mmHg for both of these groups). If the VP_ICU_ group had significantly higher UCV model scores, we would take this as evidence that some VP_ICU_ patients should have received ED vasopressors and that there was delayed vasopressor initiation, relative to the institution's usual care.

*Application of the UCV model to identify and analyze patient outliers*—We used the model to identify a cohort of patients who had vasopressors started either substantially earlier or substantially later than was predicted by the UCV model. First, we selected a numerical cut-off for the UCV model that provided high (90%) specificity for the initiation of vasopressors. Next, we applied the UCV model to all patients and to all time-points with documented hypotension. Next, we identified patients who had vasopressors started before ever crossing this threshold (i.e., earlier than predicted by the UCV model). We likewise identified patients who had vasopressors started at least 60 min after crossing this threshold (i.e., later than predicted by the UCV model). For illustration purposes, we compared one fundamental clinical characteristic of these outlier patients, which was the amount of IVF administered.

### 2.6. Statistical Analysis

Univariate comparisons used the chi-squared test for categorical variables and the Mann-Whitney-U test for continuous variables. Values of variables at different times were compared by the Kruskal-Wallis test with *post-hoc* Mann-Whitney-U test. AUCs were compared by DeLong's method (13). Empirical distribution functions of model scores were compared with the Kolmogorov-Smirnov test. All tests were two-tailed with significance at 0.05. For visualization purposes, empirical distributions were smoothed by Gaussian kernel density estimation (11). When providing information on durations and fluid volumes, we report the medians and interquartile ranges (IQRs) of the underlying cohort distributions. All computational analyses were performed in Python using the Scikit-learn (14), Scipy (15), and Statsmodels (16) libraries.

## 3. Results

### 3.1. Cohort

Of 185,949 total adult ED patient encounters during the study period, 705 met inclusion criteria for chart review, and 589 met criteria for the study cohort, of which 384 received ED vasopressors for any duration (Figure 2). Cohen's Kappa ranged from 0.85 to 0.89 for determining membership in the five outcome groups. The full patient characteristics, broken down by ED vasopressor and Non-ED vasopressor cohorts are provided in Table A1. The training set used for UCV model development included 365 patient encounters, of which 213 were in the VP_>24_ group (after exclusion of patients without a valid *t*_*f*_ due to a lack of documented hypotension prior to vasopressor administration) and 152 in the Non-VP group.

### 3.2. Development and Validation of the UCV Model for Vasopressor Initiation

The two-stage model building process selected six covariates for the final UCV model (Table 1). Laboratory measurements like lactate, creatinine, and white blood cell count did not enter the model. For example, neither the most recent nor maximum lactate value was selected, nor did univariate analysis show a difference in the first lactate between non-ED and ED vasopressor cases (Table A1). Measures of fluid responsiveness (such as changes in SBP after IVF administration or total duration with SBP < 90 mmHg after initiation of two liters of IVF) were also not selected.

**Table 1:** Final logistic regression model, with means and standard deviations from the training set for the selected variables (used for standardization in model development) provided as a reference.

**Variable**	**Mean**	**Odds ratio**	***P*-value**
	**(Standard dev.)**	**(95% CI)**	
Exp. weighted	21 (4.7) min^−1^	1.55 (1.05 – 2.29)	0.029
respiratory rate		per 5 min^−1^	
Fluids given while	890 (1200) mL	1.41 (1.04 – 1.93)	0.028
SBP <90 mmHg		per 1000 mL	
Elapsed time from	6.4 (6.1) hours	0.40 (0.27 – 0.60)	<0.001
triage		per 1 hour	
Minimum GCS	13 (3.7)	0.40 (0.26 – 0.62)	<0.001
		per 1 unit	
Minimum SpO_2_	92 (5.7) %	0.63 (0.42 – 0.94)	0.025
		per 5%	
SBP	80.0 (8.6) mmHg	0.10 (0.06 – 0.18)	<0.001
		per 5 mmHg	

*Exp. Weighted, exponentially weighted*.

The ROC AUC for the final UCV model, using leave-one-out cross-validation and applied to time-point *t*_*f*_, was 0.91 (95% CI 0.88–0.94) in the training set (*n* = 328) and 0.78 (95% CI 0.58–0.99) when applied to the full held-out validation set (*n* = 37). When evaluated at all hypotensive observations from triage through *t*_*f*_, the UCV model achieved a ROC AUC of 0.80 (95% CI 0.78–0.83) in the training set (*n* = 1,628 observations) and 0.77 (95% CI 0.68–0.86) in the validation set (*n* = 201 observations). Considering that ROC AUCs of 0.7–0.8 are generally considered acceptable and from 0.8 to 0.9 are considered excellent (17), these results supports our overarching hypothesis: a single statistical model can represent the usual care in terms of initiating vasopressors for septic shock.

**Figure 3:**
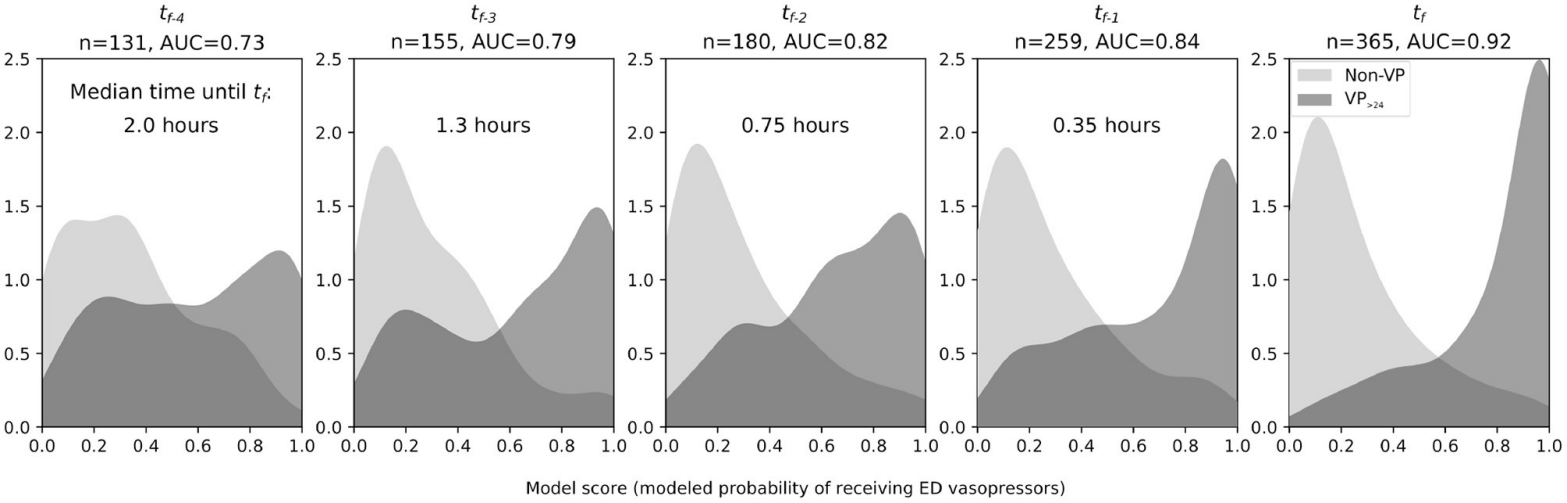
Smoothed observed density functions for scores from an LR model trained to discriminate between the VP_>24_ group (hypotensive, septic patients requiring vasopressor infusions for >24 h, dark gray) and the Non-VP group (hypotensive, septic patients not receiving vasopressors for at least 48 h, light gray). Time is referenced from the “final ED decision point” (*t*_*f*_), either just before vasopressors were initiated or just before ED hypotension resolved. From left to right: distributions at four vital signs observations (median 2.0, IQR 1.2–2.7 h) prior to *t*_*f*_, at three observations (median 1.3, IQR 0.81–2.1 h) prior to *t*_*f*_, at two observations (median 0.75, IQR 0.46–1.3 h) prior to *t*_*f*_, at one observation (median 0.35, IQR 0.18–0.68 h) prior *t*_*f*_, and at *t*_*f*_. The progressive separation of the two curves and increasing AUC show that discriminative ability increases in the approach to *t*_*f*_. However, a clear subset of ED vasopressor patients has model scores as early as *t*_*f*−4_ and *t*_*f*−3_ that exceed nearly all the scores of the Non-VP group. AUC, area under the receiver operating characteristic curve; ED, emergency department; *n*, total number of patient encounters included.

Distributions of UCV model scores evaluated at *t*_*f*−4_ through *t*_*f*−1_ showed that discrimination between VP_>24_ and Non-VP patients improved as the time approached *t*_*f*_ (Figure 3). At *t*_*f*−4_ (median 2.0 h before *t*_*f*_), the AUC was 0.73 and by *t*_*f*−1_ (0.35 h before *t*_*f*_) 0.84. Among the predictors, only SBP changed significantly over time and only in the VP_>24_ group at *t*_*f*_ (*P* < 0.001 for pairwise comparisons with all other observation times in the VP_>24_ group).

When the UCV model was applied to earlier time points and compared with alternative models that were trained specifically to those earlier time points, there were no significant differences in terms of AUC for *t*_*f*−1_, *t*_*f*−2_, and *t*_*f*−3_ (Table 2). Only at *t*_*f*−4_ did an alternative model (AUC = 0.85) significantly outperform the UCV model (AUC = 0.73, *P* < 0.01); at all other times, the UCV model was non-inferior.

**Table 2:** AUCs for each alternative model evaluated at the time of training via leave-one-out cross-validation (LOOCV) and statistical comparison with the UCV model.

**Observation**	**AUC of UCV model**	**AUC of alternative model**	***P*-value**
	**(95% CI)**	**(95% CI)**	
*t*_*f*−4_	0.73	0.85	0.003
	(0.64, 0.81)	(0.78, 0.91)	
*t*_*f*−3_	0.79	0.81	0.47
	(0.71, 0.86)	(0.74, 0.88)	
*t*_*f*−2_	0.82	0.86	0.17
	(0.75, 0.89)	(0.80, 0.92)	
*t*_*f*−1_	0.84	0.86	0.20
	(0.79, 0.89)	(0.81, 0.91)	
*t*_*f*_	0.91	0.91	
	(0.88, 0.94)	(0.88, 0.94)	

The overall composition of the UCV model vs. the models trained for earlier time points was similar (Table 3). All models selected a core set of vital signs features (including ones derived from each of GCS, SBP, and either respiratory rate or temperature) plus elapsed time from triage and a feature related to IVF administration.

**Table 3:** Final compositions of models trained at earlier observation times, with number of available encounters noted.

***t*_*f*−4_(*n* = 131)**	***t*_*f*−3_(*n* = 155)**	***t*_*f*−2_(*n* = 180)**	***t*_*f*−1_(*n* = 259)**	***t*_*f*_(*n* = 375)**
Exp. wght. temp.	Exp. wght. temp.	CHF or CKD	Exp. wght. temp.	Exp. wght. resp. rate
Time from triage	Time from triage	Exp. wght. temp.	Fluids SBP <90	Fluids SBP <90
Max pain level	Min. GCS	HR	Time from triage	Time from triage
Min. GCS	Non-white race	Time from triage	Min. GCS	Min. GCS
Min. SBP	SBP	Max. HR	Min. SpO_2_	Min. SpO_2_
Non-white race	Total fluid vol.	Min. GCS	SBP	SBP
Total fluid vol.	Urinary complaint	SBP	Total fluids	

### 3.3. Application of the “Usual Care” Dynamic Practice Model to Compare Patient Cohorts and Characterize Individual Patient Outliers

**Figure 4:**
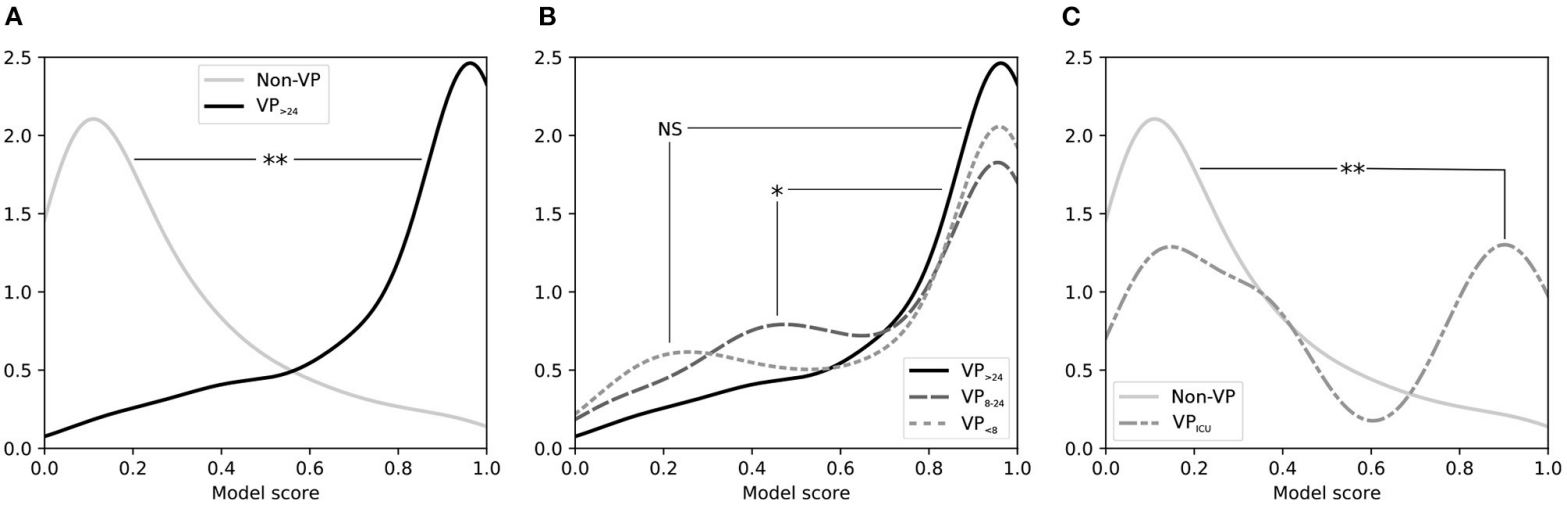
Smoothed distribution of scores for *t*_*f*_ from an LR model trained to discriminate between ED patients with sepsis requiring vasopressors and patients not requiring vasopressors. **(A)** Comparing distributions for Non-VP patients (those not given vasopressors in the first 48 h of the hospital visit) vs. VP_>24_ patients (those receiving vasopressors beginning in the ED and continuing for > 24 h) shows that the model separates these two groups well. **(B)** Comparing distributions for VP_>24_ patients vs. VP_ <8_ patients (those receiving vasopressors beginning in the ED but for < 8 h) shows that patients who were weaned from ED vasopressors quickly appeared similar to patients who needed vasopressors for a lengthy duration, though VP_8−24_ patients (those with an intermediate vasopressor duration of 8–24 h) showed a minor but statistically significant difference. **(C)** Comparing distributions for Non-VP patients (those not requiring vasopressors) vs. VP_ICU_ patients (those who received vasopressors initiated in the ICU after leaving the ED) shows that the latter had a bimodal distribution when in the ED, which was significantly higher than the Non-VP group. ED, emergency department; *t*_*f*_: “final ED decision point.”

We tested whether septic patients who were quickly (≤ 24hrs) weaned from ED vasopressors had their vasopressors initiated as per usual care. Based on the model outputs at *t*_*f*_, there was no evidence that decision-making to initiate vasopressors was any different for VP_ <8_ vs. for VP_>24_ groups: their model output distributions were not significantly different (Figure 4). There was evidence that the VP_8−24_ group was statistically different from the VP_>24_ group in terms of model output (*P* < 0.05), but upon inspection, this difference appears trivial (also Figure 4).

We also tested whether septic patients who did not have vasopressors started in the ED but did have vasopressors started shortly after ICU admission had their vasopressors initiated as per usual care. The UCV model output distribution for VP_ICU_ showed a distinctly bimodal distribution that was statistically different from Non-VP patients (*P* < 0.001), with one peak representing patients with low model scores (i.e., did not appear to need vasopressors in the ED and did not receive vasopressors in the ED), and a second peak representing high model scores (i.e., did appear to need vasopressors in the ED, but only received vasopressors after arriving in an ICU); see Figure 4.

Finally, to illustrate how the dynamic model can be used to identify and characterize a cohort of patients who did not receive usual care, we applied the UCV model to all patients (including all VP_>24_, VP_8−24_, VP_ <8_, VP_ICU_, and Non-VP) at all hypotensive time points over their entire ED stays. Using a model threshold of 0.80, which achieved a 90% specificity across all hypotensive observations, 283 vasopressor patients met this threshold at least once prior to vasopressor initiation, for a patient-level sensitivity of 74% and positive predictive value of 88%.

We examined patients who had vasopressors started without reaching the model threshold. The majority of these had vasopressors started in their first hour after ED arrival, often before there was documented completion of fluid administration.

**Figure 5:**
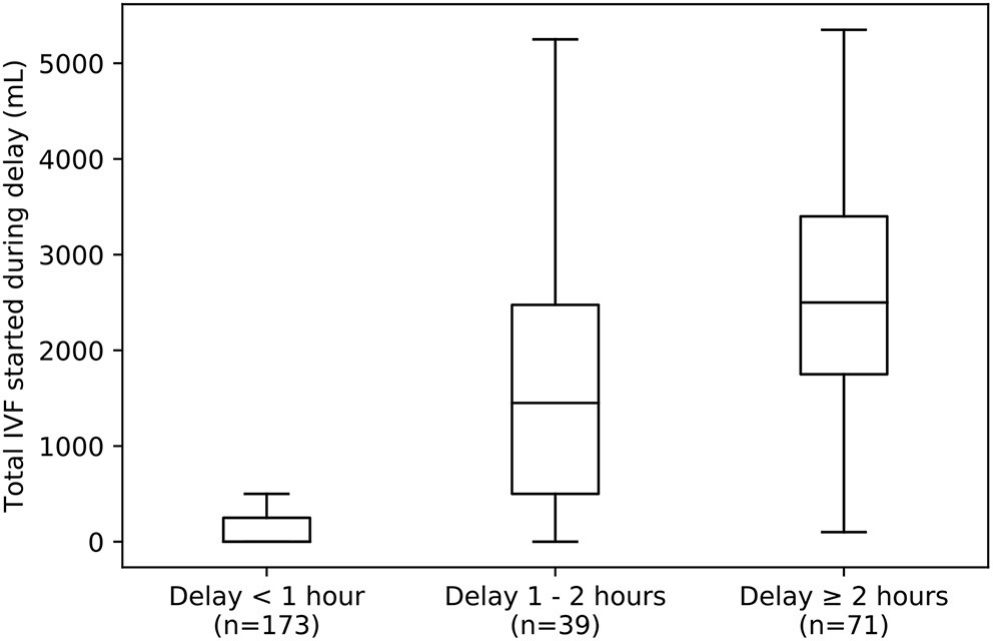
Box-plot summarizing distributions of IVF volumes given to VP_>24_ patients during delays of varying lengths between reaching a UCV model threshold of 0.8 and initiation of vasopressor therapy. Left: Most ED patients receive vasopressors within 1 h after reaching this threshold (*n* = 110 out of 283 VP_>24_ patients who reached a threshold of 0.8) and receive little IVF in that time (median 0 mL, IQR 0 – 250 mL). Middle and Right: However, a large minority experience delays of 1 to 2 h (middle, *n* = 39) or even greater than 2 h (right, *n* = 71), and these patients tend to receive large IVF volumes (median 1,450 mL, IQR 500–2,475 mL for those with delays of of 1 to 2 h, and median 2,500 mL, IQR 1,750–2,400 mL for those with delays of 2 h or more). IVF, intravenous fluid.

Seventyfour percent of ED vasopressor patients did reach the model threshold of 0.80; the median time between reaching the high-specificity threshold and actual vasopressor initiation was 0.52 h. When this time was below 1 h, very little IVF was administered in that interim period (median 0 mL, IQR 0 – 250). However, this time exceeded 1 h in 39% of vasopressor patients who met the threshold of 0.80. In this subset of patients substantial IVF volumes were administered (median 2,250 mL, IQR 1,200–3,300) during that interim period (Figure 5).

## 4. Discussion

Our analysis found that a statistical “dynamic practice” model based on a small number of clinical factors can reasonably model the decision to begin or abstain from vasopressors in hypotensive septic patients. The model was effective at discriminating between VP_>24_ and Non-VP at the time point at which vasopressors were initiated or hypotension resolved. It also offered significant discriminatory ability when applied across all prior time points. Not only did we find, as expected, that depth of hypotension and administered IVF volume were important to the model, but other metrics of disease severity were as well (Table 1). Specifically, four predictors were basic vital signs, including SpO_2_ and the three components of the qSOFA sepsis severity score. IVF volume was a fifth factor: the more IVF a hypotensive patient had already received, the more likely that vasopressors would be required to resolve ongoing hypotension. This implies that, at our medical center, usual care may not be as simple as “liberal fluids” as has been previously described (6): we found that clinicians typically titrated their decision to initiate vasopressors to overall disease severity (*e.g*., qSOFA components). Interestingly, laboratory measurements like lactate, creatinine, and white blood cell count did not enter the model.

Before patients were started on vasopressors, the UCV model showed that they had lower model scores at *t*_*f*−4_ through *t*_*f*−1_ compared to at *t*_*f*_ (Figure 3). In other words, at time points before the initiation of vasopressors, VP_>24_ patients' clinical parameters were typically *not consistent* with vasopressor initiation. A test of within-group temporal changes in the model predictor variables showed that it was only SBP that changed significantly across the time steps leading up to *t*_*f*_.

Our analysis of discrimination at times before *t*_*f*_ (i.e., comparing the primary UCV model vs. alternative models trained specifically for *t*_*f*−1_, *t*_*f*−1_, *t*_*f*−3_, and *t*_*f*−4_) showed that the UCV model was itself generally valid at earlier times, achieving similar performance to the alternative models, while selecting very similar feature sets. Taken together, these findings suggest that, in usual care, the decision to initiate vasopressors is titrated to disease severity: the probability of starting vasopressors at any given time increased as overall disease severity increased (as measured by metrics, such as GCS, respiratory rate, SBP, and SpO_2_). Conversely, patients with isolated hypotension in the absence of other signs of organ dysfunction were less likely to have vasopressors initiated.

*Application of the “Usual Care” Dynamic Practice Model to Compare Patient Cohorts*—After developing and validating the UCV model, we used it to compare subject cohorts. The distribution of UCV model scores of the VP_ <8_ group was not significantly different from that of the VP_>24_ group (Figure 4). While there was evidence that the VP_8−24_ group was statistically different from the VP_>24_ group in terms of model output (*P* < 0.05), this difference appears clinically unimportant.

By contrast, there was evidence that a subset of patients who had vasopressors started in the ICU had delayed initiation in the ED, compared with usual care. The UCV model output distribution for VP_ICU_ showed a distinctly bimodal distribution that was statistically different from Non-VP patients (*P* < 0.001). The peak with high UCV model scores represents the subset of patients who ordinary would have had vasopressors in the ED. Although this finding is markedly apparent in Figure 4 and is statistically significant, it represents a small number of actual patients (only a subset of the 53 patients comprising the VP_ICU_ group). For these patients, perhaps the ED team felt that the vasopressor initiation could be safely deferred until the patient arrived in the ICU.

These analytic exercises illustrate how dynamic care modeling can be applied to test for differences in dynamic clinical decision-making between cohorts or populations. This tool can be used for analyzing and interpreting clinical trials. As discussed in the Introduction, there has been substantial controversy regarding the CLOVERS trial about how to define usual care (6, 7). We suggest that a dynamic care model is an objective method for addressing such controversies: dynamic care in one population or cohort can be modeled, after which the model can be applied to another population, to objectively test whether care (in this case, initiation of vasopressors) is significantly different.

*Application of the “usual care” dynamic practice model to identify a subset of patients who did not receive usual care*—To illustrate how the dynamic model can be used to identify and characterize a subset of patients who did not receive usual care, we applied the UCV model to all patients (including all VP_>24_, VP_8−24_, VP_ <8_, VP_ICU_, and Non-VP) at all hypotensive time points over their entire ED stays. According to this methodology, the majority of VP_>24_ patients had no evidence of delayed vasopressors. Moreover, for Non-VP patients, who never received vasopressors, there was strong agreement between modeled and observed practice. Patients who had persistently lower scores per the UCV model throughout the ED stay rarely received vasopressors in the subsequent 48 h.

In terms of care that was outside of the usual care, we found that 25% of the vasopressor patients had initiation earlier than the model predicted. The vast majority of these were started on vasopressors very early in their ED care, before any documented IVF administration. This suggests that either there was a documentation issue (i.e., imprecise documentation for patients who arrived to the ED and received a series of rapid interventions) or, alternatively, that there were other clinical cues used to decide on immediate vasopressors before any IVF.

Most ED vasopressor patients (61%) who met the model threshold for “vasopressor initiation” had vasopressors initiated within an hour of reaching the threshold (Figure 5). However, for the 39% who had at least 1 h pass between threshold-crossing and vasopressor initiation, that delay was associated with additional IVF. Those with 1–2 h of delay received a median of 1500 mL (IQR 1000–2475 mL) of fluid during that time, and those with 2 or more hours received 2500 mL (1800–3400 mL). This might have been a cohort treated with “liberal fluids” (6). Alternatively, this might represent “clinical inertia” [a phenomenon studied in more detail in chronic disease management (18)] in continuing fluid resuscitation rather than altering course to use vasoactive therapy. In short, application of the model was able to identify a sizable minority of vasopressor patients who may have received excess IVF, instead of vasopressor initiation, consistent with the institution's usual care. Similarly, upon expansion to multi-center application, such usual care model might be able to identify care differences between centers and thereby help provide evidence regarding the use of liberal vs. restrictive fluid administration (19).

*Limitations*—First, the final dynamic practice model and all associated findings arose form a single center. “Usual care” likely varies by institution. However, the model did contain the three parameters of the qSOFA score (3), suggesting that the model was discriminating between vasopressor and non-vasopressor patients on the basis of established metrics of overall sepsis severity; this finding is likely to be externally valid. As described above, the dynamic practice model can be readily applied to data from other centers or other patient cohorts to objectively test whether the care was significantly different or not.

Second, the model and analysis focused on objectively characterizing “usual care” and not whether vasopressors were beneficial for a patient. The results yielded insights into clinician behavior and an objective tool for evaluating when patients received management consistent with usual care and when management was atypical. Further work is necessary to evaluate whether deviations from usual care are associated with different patient outcomes (the current dataset is underpowered for outcome analysis).

Third, the UCV model made use of one variable, elapsed time from triage, of which we had no clear *a priori* reason to expect inclusion. This may indicate a survivorship bias, where patients receiving vasopressors tended to have a *t*_*f*_ earlier than those not receiving vasopressors due to our definition of *t*_*f*_, and inclusion of this variable allows the model to adjust for this. Additionally, we note that hypotension tended to start earlier for VP_>24_ patients vs. Non-VP patients (0.9 h vs 2.0 h after triage, respectively), suggesting that late-developing hypotension may be more benign than early hypotension.

In summary, we found that the decisions made by clinicians to begin or forego vasopressors in hypotensive sepsis patients can be well modeled by a small number of clinical factors. This model included variables directly related to hemodynamic management (depth of hypotension and administered IVF volume), as expected, as well as other established metrics of disease severity. This demonstrates the possibility of using basic clinical parameters to model the decision-making by which vasopressors are initiated at a given time point and directly links a patient's clinical state to a diagnosis of septic shock according to the Sepsis-3 definition. As demonstrated in this report, this model offers a tool for comparing whether usual care was consistent between two populations or atypical within any subpopulations. The model's strong discriminative performance also suggests further potential to create data-driven tools as real-time aids for clinical decision-making.

## Data Availability Statement

The datasets presented in this article are not readily available because release of the data is subject to oversight by the Partners data oversight committee. Requests to access the datasets should be directed to Dr. Andrew Reisner, areisner@mgh.harvard.edu.

## Ethics Statement

The studies involving human participants were reviewed and approved by the Institutional Review Board of the Massachusetts General Hospital. The Ethics Committee waived the requirement of written informed consent for participation.

## Author Contributions

VP: conceptualization, model development, data analysis, statistics, manuscript drafting and revision, and approval of final manuscript. AR and TH: conceptualization, model development, data review, manuscript revision, and approval of final manuscript. JL: database development, data review, and approval of final manuscript. MF: data review, manuscript revision, and approval of final manuscript. All authors contributed to the article and approved the submitted version.

## Funding

This work was supported in part by a National Defense Science and Engineering Graduate Fellowship, the MIT-MGH Grand Challenge in Diagnostics, and the CRICO Risk Management Organization. The authors state that this study received funding from Nihon Kohden Corporation. Nihon Kohden Corporation was not involved in the study design, collection, analysis, interpretation of data, the writing of this article or the decision to submit it for publication.

## Conflict of Interest

The authors declare that the research was conducted in the absence of any commercial or financial relationships that could be construed as a potential conflict of interest.

## Publisher's Note

All claims expressed in this article are solely those of the authors and do not necessarily represent those of their affiliated organizations, or those of the publisher, the editors and the reviewers. Any product that may be evaluated in this article, or claim that may be made by its manufacturer, is not guaranteed or endorsed by the publisher.

## Footnotes

^1^Per the Sepsis-3 definition: “Patients with septic shock can be identified with a clinical construct of sepsis with persisting hypotension requiring vasopressors to maintain MAP ≥65 mm Hg and having a serum lactate level ≥2 mmol/L [18mg/dL] despite adequate volume resuscitation” (3).

^2^The Surviving Sepsis Campaign advises: “[i]f blood pressure is not restored after initial fluid resuscitation, then vasopressors should be commenced within the first hour to achieve mean arterial pressure (MAP) of ≥65 mmHg” (4, 5), whereas repeated IVF boluses are advised—with a “weak recommendation”—when dynamic measures indicate patients are likely to experience an increase in stroke volume with additional fluids.

^3^In a preliminary analysis, we studied all time points (not just hypotensive time points). This was a trivial problem: vasopressors were never started without the occurrence of hypotension. Therefore, the current report focused on the more interesting question of predicting vasopressor initiation during hypotension.

## Appendix

Table A1 lists the patient characteristics, broken down by patients who did receive vasopressors in the emergency department (ED-vasopressors) and those who did not receive vasopressors in the emergency department (Non-ED vasopressors). Compared with patients who did not receive vasopressors, ED vasopressor patients were slightly older (median 66 vs. 64 years, *P* = 0.014) and had greater incidences of certain comorbidities, including coronary artery disease (25 vs. 16%, *P* = 0.010), congestive heart failure (27 vs. 16%, *P* < 0.01), and chronic kidney disease (28 vs. 20%, *P* = 0.039), while receiving less ED IVF (3,600 vs. 4,100 mL, *P* < 0.001). ED vasopressor patients also had greater SOFA scores (median 9 vs. 4, *P* < 0.001) with more frequent direct admission to an ICU (91 vs. 39%, *P* < 0.001) and hospital mortality (29 vs. 13%, *P* < 0.001).

**Table A1:** Clinical characteristics of the study cohort, compared across patients receiving vasopressors in the ED and patients not receiving vasopressors in the ED.

**Variable, units**	**Non-ED vasopressor pts**.	**ED vasopressor pts**.	**P-value**
	**(*N* = 205)**	**(*N* = 384)**	
Age, years	63 (49 – 75)	66 (55 – 77)	0.014^*^
Male, %	50	58	0.056
Non-white, %	20	18	0.57
Triage GCS score	15 (15 – 15)	15 (13 – 15)	<0.001^*^
Triage respiratory rate,	20	20	0.016^*^
min^−1^	(18 – 22)	(18 – 24)	
Triage SpO_2_, %	97	96	0.064
	(94 – 98)	(93 – 98)	
Triage temperature, °F	98.2	98.1	0.22
	(97.2 – 99.9)	(97.1 – 99.4)	
First serum lactate	2.8	2.8	0.55
mmol/L	(1.5 – 4.5)	(1.8 – 4.5)	
Serum BUN, mg/dL	23	30	<0.001^*^
	(15 – 40)	(18 – 49)	<0.001^*^
Serum creatinine,	1.3	1.6	<0.001^*^
mg/dL	(0.88 – 2.1)	(1.1 – 2.7)	
Platelet count,	191	192	<0.001^*^
1000/μL	(137 – 282)	(111 – 264)	0.15
White blood cell count	12.5	13.3	0.69
1000/μL	(7.3 – 18.2)	(7.0 – 19.3)	
Cancer, %	24	25	0.79
Coronary artery disease,	16	25	0.010^*^
%			
Congestive heart failure,	16	27	<0.001^*^
%			
Chronic kidney disease,	20	28	0.039^*^
%			
Chronic obstructive	17	20	0.36
pulmonary disease, %			
Cerebrovascular	7.8	12	0.18
accident, %			
Diabetes, %	23	32	0.036^*^
Liver disease, %	8.8	6.8	0.47
Source, %
Pulmonary	22	26	
Urinary	22	19	
Intra-abdominal	25	23	
Skin/soft tissue	6.3	6.3	
Unknown	25	25	
Other	3.9	5.7	
Total IVF volume,	4100	3600	<0.001^*^
started, mL	(3050 – 5500)	(2300 – 4800)	
SOFA score	4 (3 –6)	9 (7 – 11)	<0.001^*^
Hospital mortality, %	13	29	<0.001^*^
Direct ICU admission, %	39	91	<0.001^*^

*Values are median (IQR) or fraction of cohort*.

* *P < 0.05*.
